# Mechanotransduction in T Cell Development, Differentiation and Function

**DOI:** 10.3390/cells9020364

**Published:** 2020-02-05

**Authors:** Muaz Rushdi, Kaitao Li, Zhou Yuan, Stefano Travaglino, Arash Grakoui, Cheng Zhu

**Affiliations:** 1 Wallace H. Coulter Department of Biomedical Engineering, Georgia Institute of Technology, Atlanta, GA 30332, USA; muaz.rushdi@gmail.com (M.R.); kaitaoli@gatech.edu (K.L.); stefano.travaglino@gatech.edu (S.T.); 2 Parker H. Petit Institute for Bioengineering and Bioscience, Georgia Institute of Technology, Atlanta, GA 30332, USA; zyuan8@gatech.edu; 3 George W. Woodruff School of Mechanical Engineering, Georgia Institute of Technology, Atlanta, GA 30313, USA; 4 Emory Vaccine Center, Division of Microbiology and Immunology, Yerkes Research Primate Center, Emory University School of Medicine, Atlanta, GA 30329, USA; arash.grakoui@emory.edu; 5 Division of Infectious Diseases, Department of Medicine, Emory University School of Medicine, Atlanta, GA 30322, USA

**Keywords:** T cell antigen receptor, lymphocytes, thymocytes, catch bond, force, stiffness

## Abstract

Cells in the body are actively engaging with their environments that include both biochemical and biophysical aspects. The process by which cells convert mechanical stimuli from their environment to intracellular biochemical signals is known as mechanotransduction. Exemplifying the reliance on mechanotransduction for their development, differentiation and function are T cells, which are central to adaptive immune responses. T cell mechanoimmunology is an emerging field that studies how T cells sense, respond and adapt to the mechanical cues that they encounter throughout their life cycle. Here we review different stages of the T cell’s life cycle where existing studies have shown important effects of mechanical force or matrix stiffness on a T cell as sensed through its surface molecules, including modulating receptor–ligand interactions, inducing protein conformational changes, triggering signal transduction, amplifying antigen discrimination and ensuring directed targeted cell killing. We suggest that including mechanical considerations in the immunological studies of T cells would inform a more holistic understanding of their development, differentiation and function.

## 1. Introduction

Progenitor cells must receive specific cues upon encountering their niche to ensure appropriate development into mature cells and to differentiate into specific cell types [1,2]. While some cues come in the form of biochemical signals such as receptors binding to soluble cytokines, other cues are physically felt by cells. Examples of the latter cues include but are not limited to the stiffness or roughness of the extracellular matrix (ECM), pressure by adjacent cells, and forces on bonds of receptor–ligand pairs on opposing surfaces [3,4,5]. These considerations are biomechanical in nature as opposed to biochemical where no forces are exerted on or felt by the receptor after binding to a soluble cytokine. Like nonliving objects, mechanical stimuli on biological cells and molecules can cause mechanical responses, such as motion, deformation and conformational changes [6]. The field of biomechanics studies such causal relationships through the measurements of mechanical properties of cells and molecules and by application of the principles of mechanics. By comparison, mechanobiology studies how cells sense, respond to, and adapt to changing mechanical cues in their environment by biological means.

**Figure 1:**
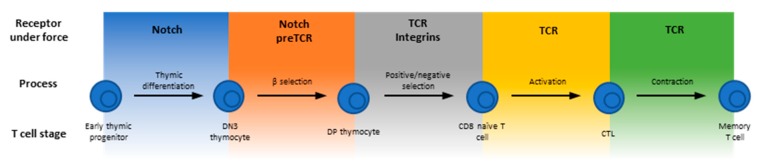
Mechanotransduction occurs throughout the life cycle of T lineage cells. Schematic depicting development of a T lineage cell and instances where receptors on the developing cell have been studied to be subject to forces. Lymphoid progenitor encounters Notch and commits to a T cell lineage in the thymus; at the DN3 stage of thymocyte development, the pre-T cell antigen receptor (TCR) may bind to pMHC under force; at the developmental stage from double positive (DP) to single-positive (SP) thymocytes, migration from cortex to medulla is controlled by force-modulated ligand dissociation of β_1_ integrins; the αβTCR relies on force to discriminate between positive and negative selection ligands; the naïve CD8^+^ T cell is activated when TCR is triggered by pMHC under force; memory T cells survive the contraction phase in part through TCR signaling.

As part of the Cells’ Special Issue on “Mechanotransduction in Control of Cell Fate and Function”, this review focuses on the emerging field of T cell mechanoimmunology to exemplify the reliance on sensing and responding to the mechanical environment for the development, differentiation and function of the T lineage cells, which are central to the adaptive immune system. T cell progenitors called thymocytes develop in the thymus where they are subject to constant interrogation by their environment to proceed in their developmental pathway through various stages of Notch signaling, beta-selection, positive selection and negative selection [7,8] (Figure 1). Upon egress from the thymus, mature T cells that have not yet encountered non-self (e.g., viral) antigens, circulate the body and lymph nodes as naïve cells. Recognition of antigens from foreign or transformed proteins stimulates T cell proliferation, and the expanded antigen-specific cells patrol the body to carry out their effector functions [9,10,11]. Finally, following clearance of infected or cancerous cells, a significant contraction phase occurs whereby the vast majority of the expanded T cells undergo apoptosis possibly due to insufficient antigen stimulation, leaving only a small fraction that differentiate into memory T cells [12,13,14,15].

Many molecules are known to be involved in various stages of T cells’ life cycle. This review focuses on those that function in a mechanically stressful environment, usually experience forces, and have been suggested to potentially play some roles in T cell mechanoimmunology (Figure 1). For example, antigen recognition requires direct physical contact between the T cell and an antigen presenting cell (APC). It is determined by the interactions of the T cell antigen receptor (TCR) and the CD4 or CD8 co-receptor with the antigenic peptides presented by the major histocompatibility complex (pMHC) molecules, as well as other T cell surface adhesion and co-stimulatory/co-inhibitory molecules with their corresponding APC ligands [16,17]. These receptor–ligand bonds physically bridge the two cells and hence are inevitably subject to mechanical forces. This is because T cells and/or APCs are highly motile, their cytoskeleton is very dynamic and their membrane vigorously undulates, especially during the time when the T cell is searching for antigen on, and forming an immunological synapse (IS) with, the APC [18]. Forces accompanying cellular mechanical activities may act on the cross-junctional receptor–ligand bonds to perpetuate or retard such activities. Regardless of which is the case, the T cell would receive mechanical cues via these specific receptor–ligand interactions, which may initiate mechanotransduction.

Mechanotransduction is the process whereby a mechanical cue is transduced into a biochemical signal [6]. Related processes also include presentation, transmission and response to a mechanical cue. Molecules, parts of a molecule or assemblies of molecules that play functional roles in these processes are referred to as mechanopresenters, mechanotransmitters, mechanotransducers and mechanoresponders, respectively [6]. To carry out such functions, these molecules may undergo force-induced changes in inter- and intra-molecular interactions or in structures and conformations, which allow biochemical signaling events to occur intracellularly in response to extracellular biomechanical events [6]. At the molecular level, important questions in mechanobiology include the identification of which molecules play what roles and the mechanistic elucidation of how they work. Whereas an increasing number of molecules have been found to play some of the above functional roles, the understanding of how they work remains largely lacking. Nevertheless, many such molecules have been found to exhibit some common features: (1) exhibit dynamic bonds to allow mechanoregulation of bonding kinetics. (2) Display force-induced conformational changes for allosteric actions. (3) Are capable of decoding the information embedded in the ligand and/or in the force waveform. (4) Allow intracellular regulation of extracellular bonding. (5) Offer new insights when the mechanical effects are considered. Here, we review several areas of published work where the above features have been reported for the molecules listed in Figure 1. These examples suggest that mechanotransduction critically impacts T cell development, differentiation and function.

## 2. Early Force-Dependent Notch Signaling Directs T Cell Lineage

**Figure 2:**
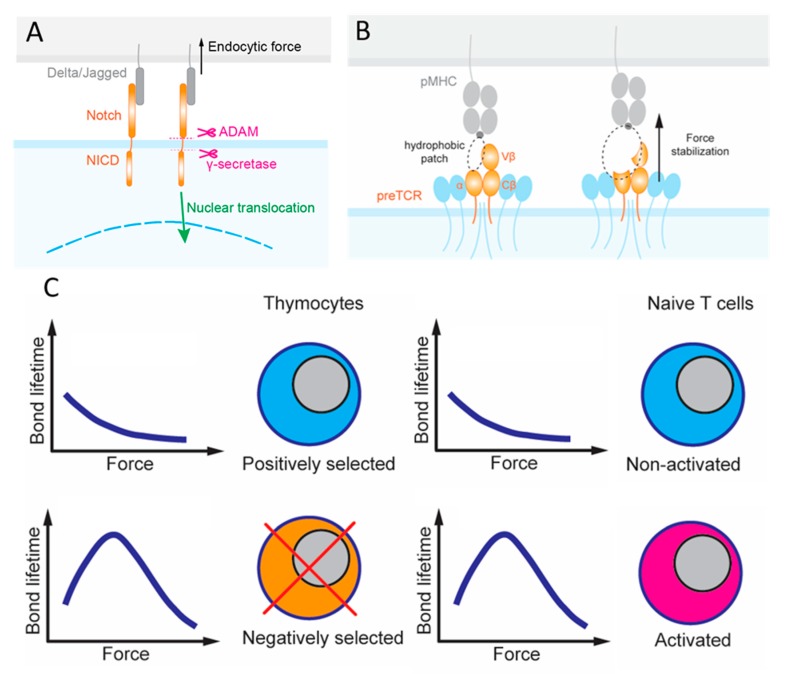
Forces induce differential outcomes at different T cell development stages. Schematic depicting four developmental stages of T cells and how receptors experience force. (**A**) Notch receptor undergoes force induced conformational changes to reveal cryptic sites for proteolytic cleavage by γ-secretase and a disintegrin and metalloprotease (ADAM) allowing for nuclear translocation of the Notch intracellular domain (NICD). (**B**) A hydrophobic patch created by Vβ and pTα allows for broad pMHC binding and is potentially force stabilized by the FG-loop of Cβ. (**C**) Similar to thymocyte selection, which relies on dynamic catch bonds to discriminate between positive and negative selection ligands, dynamic bonds can facilitate discrimination between the antigens that are stimulatory or non-stimulatory.

T cell progenitors from the bone marrow enter the thymus, which provides discrete microenvironments for both their commitment and stepwise maturation to the T lineage [7]. One such microenvironmental cue derives from various Notch ligands expressed on the surface of cells adjacent to these multipotent hematopoietic progenitors that express the Notch receptor (Figure 2A). The reliance on forces to activate Notch exemplifies the importance of mechanotransduction as Notch signaling facilitates the development, differentiation, proliferation and survival of these cells [19,20,21,22]. There are four Notch receptors (Notch1–4) and five Notch ligands (Jagged1, 2 and DLL1, 3 and 4). Each Notch receptor can bind to any of the five ligands with different affinities. Upon binding, the complex is under mechanical tension due to intercellular movements and endocytosis of the ligand-expressing cells [23]. Force-induced conformational changes in the Notch receptor reveal cryptic sites for proteolytic cleavage, which releases the Notch intracellular domain (NICD) that functions as a transcriptional cofactor [23,24,25,26]. This requirement for mechanical force to activate Notch is well known. However, Notch bonds with the different ligands likely transmit the duration and magnitude of force differently due to their distinct dynamic bond types [26]. Two approaches have been used to evaluate the force requirement to influence the degree of NICD translocation and eventually transcription: (i) the tension gauge tether (TGT) [25] and (ii) the biomembrane force probe (BFP) [27]. TGTs are DNA duplexes that are anchored to a surface on one strand and harbor the ligand on the other strand. TGTs are used to limit the endogenous force that the cell can generate and apply to the receptors–ligand bonds, since the DNA duplex would rupture when the force exceeds a threshold. The force threshold can be controlled by changing the G–C sequence content and whether the force is applied to unzip (the anchor and the ligand are on the same ends of duplex) or shear (the anchor and the ligand are on opposite ends of duplex) the DNA duplex to rupture it. In BFP, a precisely controlled force waveform is applied on the receptor via the engaged ligand via an ultrasensitive force transducer to allow for a range of mechanical measurements, including the force required to rupture the bond, the lifetime the bond endured, the elasticity of the receptor–ligand complex, and the conformational change of the molecule [28].

Ha and colleagues presented cells that fluoresce after Notch activation to surfaces of different ligands anchored by the same TGT. Resistive forces of only 4 pN was sufficient for the higher affinity ligand DLL4 to activate Notch whereas >4 pN was required for the lower affinity ligand Jagged to achieve the same degree of Notch activation [26]. Accumulation of these cross-junctional bonds and their ability to transmit force are dictated by their respective on-rate and force-dependent off-rate [28]. The BFP was utilized to measure these mechanokinetic parameters, finding that Notch dissociates from DLL4 and Jagged with a similar force-dependent off-rate, isolating the differential affinities of the two ligands to their difference in on-rates. Interestingly, Notch formed catch bonds with both DLL4 and Jagged at forces up to 10 pN, above which slip bonds were formed [26]. Catch bonds are an unusual kinetic behavior where exertion of a physical force on a molecular complex counter-intuitively prolongs its bond lifetime, which is in contrast to the ordinary slip bonds, where the force intuitively shortens bond lifetime [29,30]. The catch bond behavior is consistent with the purported conformational change suggested with its crystal structures to form a new interaction site [26]. Therefore, differential thymocyte mechanotransduction through Notch may serve as a mechanism to promote differential transcription. The Notch pathway achieves this by leveraging not only the molecules’ chemical features, including force-free kinetic rate and binding affinity, but also their mechanical features, including differential force-induced dynamic bonds and conformational changes. Consequently, a robust mechanism for specifically activated signaling is possible. Indeed, the canonical Notch mechanotransduction pathway has now been exploited to serve as a platform to design modular cell circuitry for forming regenerative multicellular structures and even increasingly targeted CAR T cell immunotherapy [31,32,33].

## 3. A Role for Force-Sensitive Ligand Recognition during Thymocyte Beta-Selection

Developing thymocytes initially lack expression of either CD4 or CD8 coreceptors (double negative, or DN, thymocytes) and can be further grouped into several stages based on other surface markers expressions [34]. In the first DN stage, signaling occurs through Notch as thymocytes reside at the corticomedullary junction of the thymus. Expression of CD25 and a surrogate preT-α chain (pTα) in place for the one seen in the αβTCR marks the second DN stage. β chain gene locus rearrangement begins in the third DN stage, which is also marked by lower expression of the adhesion molecule CD44. Completion of β-chain rearrangement and its subsequent expression result in pairing with the pTα chain and CD3 on the surface of the thymocyte. The formation of this pre-TCR complex likely drives the termination of β chain locus rearrangement and initiates the proliferation of cells that eventually express both coreceptors referred to as double positive (DP) thymocytes. This transition from DN to DP represents the fourth and last DN stage accompanied with lower CD25 expression [22,34].

Regarding the question of how the pre-TCR signals to terminate β locus rearrangement and induce proliferation of these cells, early studies proposed that the pre-TCR can function autonomously, meaning that it can signal for the survival and development of DP thymocytes without interacting with a ligand [35,36,37]. One proposed mechanism for this process, also known as beta-selection, is oligomerization of the receptors. However, NMR chemical shift titrations observed different β-chain patches recognizing their respective ligands, and micropipette adhesion frequency experiment quantified the force-free two-dimensional (2D) affinities and kinetic rates of pre-TCR interaction with a broad range of ligands, indicating the capacity of pre-TCR to bind ligands, albeit in more promiscuous fashion than mature TCR [38]. BFP and optical tweezers (OT) were used to measure the force-dependent bond lifetimes, finding catch bonds in most of these pre-TCR–pMHC interactions [38,39]. Specifically, pre-TCR formed catch bonds with ligands even if the β-chain was mutated to lower the binding affinity, but dynamic bonds were not observed for pre-TCR interacting with irrelevant β-chain (Figure 2B). OT differs from BFP in that the ligand-coated bead is constrained and controlled by a laser beam, which has higher spatial, temporal and force resolution, greater stability, but lower throughput. In addition to bond lifetime, the OT study also observed force-induced conformational changes, manifested as abrupt length increase in the pre-TCR–pMHC complexes that correlate with the biological activity of the ligands [39].

PTα expressing thymocytes transduced with specific β-chains trapped in microfluidic devices coated with pMHC underwent cell activation by fluxing calcium, while those expressing only pTα did not [38,39]. Combining the data of force-induced dynamic bonds and calcium triggering suggests that the pre-TCR function as a mechanosensor. In contrast to the autonomous pre-TCR signaling model, these mechanical data suggest that pre-TCR–pMHC interactions under force can trigger signaling to impact beta-selection. Since cells expressing mutant pre-TCR displayed similar proliferative capacities to cells expressing wild-type (WT) pre-TCR despite the weakened binding, it is possible that beta-selection accepts broad ligand specificity and may skew survival of thymocytes with a general predisposition to pMHC [38]. This may be achieved by a Vβ hydrophobic patch in the pre-TCR acting as a surrogate Vα domain to foster ligand-promiscuity [39] (Figure 2B). In this sense, mechanotransduction during beta-selection may serve as a first checkpoint to ensure functional TCR. Future work could investigate the interplay between Notch and pre-TCR signaling in the development of thymocytes since the absence of Notch precluded thymocytes from proliferation despite the expression of functional pre-TCR. Moreover, Notch is thought to contribute to autonomous signaling by promoting survival of DN3 thymocytes [7,21,38]. Following beta-selection, these DN thymocytes undergo a burst of proliferation ending with development into DP thymocytes that once again express recombination machinery to allow the process of efficient α-chain rearrangement to begin [40]. Future studies could also delineate the contributions of pTα against the rest of the pre-TCR complex to enable comparison to the complete αβTCR complex, with the goal of providing insight to a better-defined role for thymocyte mechanotransduction in positive selection.

## 4. PlexinD1-Sema3e Axis Regulates Integrin Catch Bonds to Impact Thymocyte Migration

Interestingly, the impact of intracellularly regulated, force-induced dynamic bonds on thymocyte development has been observed in a completely different scenario. Here the receptor–ligand pair in question is an integrin α_4_β_1_ binding to vascular adhesion molecule 1 (VCAM-1) and the cellular process involved is migration. As adhesion receptors for ligands on ECM or other cells, integrins power cell migration when they provide appropriate level of adhesiveness but impede cell migration when they adhere too strongly [41,42]. Integrins’ ligand binding activity is regulated bidirectionally by both inside-out and outside-in signaling. For example, α_IIb_β_3_, the first integrin to be discovered, have low binding activity on resting platelets but can be upregulated by inside-out signaling biochemically through soluble agonist binding to their receptors or biomechanically through surface-bound von Willebrand factor (VWF) binding to and exerting durable force on platelet glycoprotein Ibα (GPIbα), and by outside-in signaling through ligand binding to and pulling on α_IIb_β_3_ itself [43]. The upregulation of binding activity includes not only the increase in binding affinity and the prolongation in bond lifetime, but also the conversion from slip bond to catch bond or the formation of a more pronounced catch bond. Many integrins family members have been suggested to function as mechanosensors, playing crucial roles in mechanotransduction of various cell types [44,45].

Interestingly and in sharp contrast, in the context of thymocyte development, the regulatory mechanism involves a semaphorin, sema3E, binding to a plexin, plexinD1, and the effect is suppression of catch bonds [46]. DN thymocytes lacking plexinD1 differentiate in the thymic cortex into largely non-dividing DP thymocytes that display surface αβTCRs and express plexinD1, but remain sequestered in the cortex despite their high mobility [47,48]. They move toward the thymic medulla only after positive selection as a result of TCR stimulation by self-pMHC to express CD69 [49,50]. Positively selected DP cells differentiate further into CD4^+^CD8^−^ or CD4^−^CD8^+^ single-positive (SP) thymocytes. Without sema3E or by knocking out plexinD1, thymocytes became immobilized by strong adhesion of integrins α_4_β_1_ and α_6_β_1_ binding to VCAM-1 and laminin, respectively [46]. In the presence of sema3E, plexinD1-expressing SP thymocytes translocate to the medulla to complete their maturation [51,52]. BFP and flow chamber experiments demonstrated that sema3E–plexinD1 binding downregulates the binding activity of α_4_β_1_ by suppressing its catch bond with VCAM-1 to shorten the bond lifetime [46]. Such a correspondence suggests that greatly shortening the integrin–ligand bond lifetime allows thymocytes to be released from the otherwise overly sticky substrate to move towards the sema3E gradient to the thymic medulla.

## 5. Force Enhances the Discriminative Power of Positive and Negative Selection

While immature thymocytes traverse the thymus from cortex to medulla, they must functionally test their fully assembled αβTCR in a process termed negative selection [34,53,54,55,56]. The autoimmune regulator (AIRE) induces the expression of proteins in all parts of the body on thymic epithelial cells to allow MHC presentation of a wide variety of self-peptides on their surface to test the TCR on each thymocyte [8,38]. Strong TCR interaction with self-pMHC triggers negative selection signals to induce apoptosis of the auto-reactive thymocyte to prevent it from entering the periphery and causing autoimmune diseases [57].

To investigate negative selection, the Palmer lab used a system of altered peptide ligands (APLs) that possess mutations in their WT antigen peptide from the OVA protein (for example, Q4 denotes amino acid, ‘Q’, to substitute ‘N’ at the 4th residue in the WT sequence SIINFEKL) to show different selection outcomes of thymocytes from transgenic mice that express a monoclonal TCR (e.g., OT1) as seen in fetal thymic organ culture (FTOC) experiments [58,59]. High levels of CD3ζζ phosphorylation, significant apoptosis of DP thymocytes, low production of SP thymocytes and triggering of calcium flux in mature T cells all indicated that Q4 and Q4R7 are negative selection ligands at any peptide dose [58,59]. T4 varied between a positive selection ligand and a negative selection ligand as the peptide dose increased, even though it is only 50% less potent than Q4R7. Meanwhile, Q4H7, Q7 and G4 were determined to be positive selection ligands based on the contrasting thymocyte outcomes [58]. However, TCR–pMHC affinity and kinetic rates measured in the absence of force both using purified TCR in the fluidic phase (i.e., the three-dimensional, or 3D, parameters) and using membrane TCR on the T cell surface (i.e., 2D parameters) show very small differences between positive and negative selection ligands, raising the question of how thymocytes could robustly discriminate ligands of similar properties to make the correct fade decision to live or die.

In addition to the TCR, the co-receptors CD4 and CD8 have a functional role in T cell activation [16,60]. Their intracellular tails associate with the lymphocyte-specific kinase Lck, with CD4 associating more Lck than CD8 [59]. Lck phosphorylates the CD3 chains, initiating the signaling cascade to result in T cell activation [16,61]. Interestingly, thymocytes from a knock-in mouse model, CD8.4, which express a chimeric co-receptor that fuses the ectodomain of CD8 with the cytoplasmic domain of CD4 [62], shift the negative selection threshold, such that the strongest positive selection ligand Q4H7 for the OT1 thymocytes becomes the weakest negative selection ligand for the CD8.4 thymocytes in the FTOC experiment [59].

Applying force to examine these negative selection ligands, Q4 and Q4R7 formed catch bonds with OT1 thymocytes below 10 and 15 pN forces above which the interactions turned into slip bonds [63]. Interestingly, when the interactions between CD8 and negative selection ligands were prevented, the catch bonds were eliminated and the remaining TCR–pMHC bimolecular interactions behaved as slip bonds across all forces [63]. Since CD8 only forms slip bonds with MHC, the catch bonds result from the synergy generated by the cooperativity between the TCR and CD8 to bind the same pMHC and to form a TCR–pMHC–CD8 trimolecular bond, which we term dynamic catch [64]. Positive selection ligands, Q4H7, Q7 and G4 formed slips bonds with OT1 thymocytes because the weaker TCR–pMHC interactions were unable to induce TCR–CD8 synergy to form a trimolecular bond even when CD8 was allowed to bind MHC [63]. Molecular stiffness measurement demonstrated the underlying bimolecular vs. trimolecular nature of the bonds exhibiting distinct force dependencies [63].

In addition, DNA-based molecular tension probe (MTP) experiment revealed that the longer bond lifetimes of the negative selection ligands correspond to the more sustained endogenous forces that thymocytes pulled on the engaged pMHCs [63]. MTP is similar to TGT in that both use the same property of a DNA duplex, which can be ruptured by a defined force. Unlike TGT, which prevents excessive force to exert on the receptor–ligand bond upon DNA rupture, MTP reports the number of receptor–ligand bonds with an above threshold force exerting on them, which unzips a DNA hairpin to separate a quencher from a fluorophore to allow fluorescence to be imaged [65]. A consequence of the different dynamic bond types is that they greatly amplify the differential bond lifetimes between the positive and negative selection ligands under 10–15 pN force. Accordingly, a model has been formulated to explain how the force-induced TCR–CD8 synergy helps resolve the competing demands on the TCR to have both high sensitivity and specificity [63].

Interestingly, the ability to induce cooperative binding to pMHC by TCR and CD8 not only depends on the peptide, but also is regulated by Lck [63]. Increasing the intracellular association with Lck using the chimeric coreceptor enhanced the ability of CD8.4 to synergize with the TCR despite its weaker interaction with Q4H7, thereby converting the extracellular bonds into cooperative TCR–pMHC–CD8.4 trimolecular catch bonds [63]. Conversely, inhibiting Lck activity reduced the ability of the coreceptor to synergize with the TCR despite its stronger interactions with Q4R7 (for OT1 thymocytes) and Q4H7 (for CD8.4 thymocytes), thereby converting bonds of OT1 thymocytes with Q4R7 as well as bonds of CD8.4 thymocytes with Q4H7 into slip bonds [63]. In addition, using a knock-in mouse model that replaces the six tyrosines by phenylalanines in the CD3ζζ to reduce the number of available immunoreceptor tyrosine-based activation motifs (ITAMs) in the TCR-CD3 complex for Lck to phosphorylate impaired negative selection and decreased the force range of TCR–pMHC–CD8.4 trimolecular catch bonds [63,66]. This intracellular regulation of extracellular binding of a receptor resembles the inside-out signaling seen in integrins.

These results underlie a model whereby initial *trans*-interaction of TCR with negative selection pMHC triggers binding and phosphorylation of CD3 by the intracellular kinase Lck, which also serves as an adaptor to connect CD8 to TCR (both via *cis*-interactions). The initial signaling induces thymocyte forces, which prolong bond lifetimes by inducing cooperativity between TCR and CD8 to bind pMHC synergistically (via *trans*-interaction again), forming a trimolecular catch bond. These *trans*- and *cis*- heterodimeric interactions form a loop of self- and cross-reinforcement, thereby enabling sufficient signaling to induce negative selection. Such a mechanism for bond lifetime prolongation and signaling enhancement depends on the forces exerted on the TCR–pMHC–CD8 bonds and applied by the thymocytes, underscoring the importance of mechanotransduction in thymocyte development and survival (Figure 2C). Indeed, all five mechanotransduction features were encountered when studying this heterodimeric interaction: Thymocytes exert endogenous forces on the receptor–ligand bonds; force modulates receptor–ligand kinetics to exhibit dynamic bonds; possible Lck conformational changes enable the heterodimeric interaction; the TCR decodes the peptide contents with single amino acid resolution for the purposes of selection; intracellular Lck binding influences extracellular heterodimer formation and by studying this system, new insights were gained regarding how force amplifies peptide discrimination without sacrificing sensitivity of the TCR. Future studies will define detailed mechanotransduction mechanisms that translate amplification of bond lifetimes by negative selection ligands into biochemical signaling cascades that induce apoptosis. Additionally, while the formation of dynamic bonds was proposed as a mechanism to distinguish selection processes, it is unclear what features in negative selection ligands initiate the heterodimeric interaction loop.

## 6. Peripheral T Cell Homeostasis and Activation

Positive selection ligands may not elicit dynamic catch with the TCR and CD8 as do negative selection ligands, but these relatively weak and transient interactions are necessary for the thymocytes to progress into the SP stage [67,68]. Without interactions with positive selection ligands the DP thymocytes would die by neglect [8]. Even after their release from the thymus, therefore, naïve T cells rely on tonic signals from self-antigens to maintain homeostasis [69,70,71]. While the characteristics of TCR interactions with and signals induced by these low-affinity ligands are not fully understood, one possible explanation for the role of self-antigens in homeostasis is that a peripheral T cell clone expressing TCR with specificity to foreign antigen may exhibit a degree of cross reactivity with self-antigens, just enough for survival but too weak to trigger T cell activation [72].

In the event that a cognate antigen is encountered, a process termed TCR triggering takes place converting the ligand binding event on the signaling domain-lacking-αβTCR to the phosphorylation of up to 10 ITAMs in the cytosolic tails of the associated CD3δε, CD3γε and CD3ζζ complexes. Ensuing signaling cascades of kinases and agglomeration of adaptor molecules lead to T cell activation, which can be measured in short-term (e.g., phosphorylation of proximal signaling proteins and intracellular calcium flux), midterm (e.g., expression of activation markers and cytokine production) and long-term (e.g., proliferation). Much of our understanding of what features of the TCR that allow it to achieve both high sensitivity and specificity in antigen recognition has come from studies in this stage of development where naïve T cells encounter foreign antigen in the peripheral lymph nodes. A common approach in these studies is to examine known TCRs expressed on cell lines or primary T cells isolated from transgenic or retrogenic animals against a panel of APLs for correlation (or the lack thereof) of the APLs’ potencies to activate T cells with chosen metrics of interactions between the APLs and the TCR. Several metrics have been used to characterize this interaction, including the docking geometry observed in the TCR–pMHC co-crystal structure [73], thermodynamic properties [74], molecular flexibility [75], force-free binding affinity and kinetic rates (previously 3D parameters and more recently 2D measurements) [76], force-dependent bond lifetime [4], endogenous force on the TCR [77] and TCR–pMHC bond conformation and the changes thereof [78,79,80].

The last three metrics above are directly relevant to mechanotransduction. Using BFP [80,81,82,83] and OT [79] to measure force-modulated bond dissociation revealed catch and slip bonds (collectively referred to as dynamic bonds) for TCR–pMHC interactions on both CD4^+^ [82] and CD8^+^ [79,80,81,83] T cells. Importantly, the dynamic bond type of these interactions can change with the peptide potency, converting from slip bonds to catch bonds as the ligand biological activity increases [79,80,81,82]. Impact of the catch and slip bond dichotomy in T cell signaling has been demonstrated when different TCR–pMHC interactions with similar 3D affinities distinguished their capacity to initiate intracellular signaling by their ability, or lack thereof, to form catch bonds [83] (Figure 2C). More directly, experiments using flow [84], OT [85,86], BFP [81] and atomic force microscopy (AFM) [87] combined with calcium imaging demonstrated that applying force to the TCR can trigger calcium signals that match the dynamic bonds [81]; note that force may not be necessary for TCR triggering, as incubating T cells with soluble anti-TCR antibody or pMHC tetramer, or contacting T cells to immobilized pMHC of very high density can also activate T cells [85]. Distinct force waveforms also exerted on single TCR show different capacities to induce calcium signaling [81,85,86]. These data indicate that the TCR can function as a mechanosensor as it can decode the information encoded not only in the ligand but also in the force waveform. However, it is unclear whether the TCR dynamic bonds occur under physiological conditions. In fact, applying linearly increasing ramp force and constant camped force would result in different dynamic bonds [88]. It is also unclear whether and, if so, how such force-modulated ligand binding property contributes to answering the TCR triggering question, i.e., how membrane distal ligand recognition can lead to intracellular signaling events on adjacent proteins, especially since TCR does not possess its own signaling domains. It should be noted that whereas exerting forces on the TCR can initiate signaling, it is also possible that forces are exerted after the TCR is triggered.

To explore the question of whether the TCR experiences endogenous force, MTP was used, showing that, within seconds of ligand binding, T cells generate actin- and myosin-dependent forces to pull on their TCRs and/or CD8s [77]. Remarkably, the 12-19 pN forces correspond to the optimal force range where the lifetimes reach maximum as the TCR bonds (with or without cooperation with CD8) with agonist pMHCs transition from catch to slip bonds [63,79,80,81,83]. Interestingly, concurrent MTP and calcium imaging revealed that force rises before, but reach maximum after, intracellular calcium flux, suggesting a positive feedback loop such that force induces calcium, and calcium reinforces force. The force signal depended on CD8 and Lck, and was enhanced by integrin α_L_β_2_ [77]. Importantly, TCR force correlated with the peptide potency and with the phosphorylation of zeta-chain-associated protein kinase 70 (ZAP-70) measured concurrently on the same cell. To assess the importance of pulling the TCR by endogenous force to T cell activation, TGT was used, showing that limiting TCR force to level below 12 pN preferentially suppressed ZAP-70 phosphorylation induced by more potent ligand, thereby diminishing the correlation between peptide potency and ZAP-70 phosphorylation [77]. Thus, endogenous force on the TCR is important not only for T cell activation but also for antigen discrimination. More recently, a new version of MTP has been developed that can be locked in the fluorescent state, thereby providing a more sensitive platform to potentially detect even the more transient TCR–pMHC interactions [89].

The effect of force direction on TCR triggering has been a topic of interest. By moving T cell on the microscope stage relative to an OT-trapped bead coated with anti-CD3 or pMHC, tangential, but not normal, movement (relative to the cell-bead interface) was found to trigger intracellular calcium flux, allowing Kim et al. to propose that the TCR is an anisotropic mechanosensor [86]. Although not a single-molecule study, Li et al. found that imposing both normal (by using micropipette aspiration) and tangential (by applying orthogonal flow) forces on T cells immobilized to surfaces coated with anti-CD3 (but not control antibody) induced calcium flux [84]. By comparison, BFP experiments found that pulling on TCR–pMHC bonds with constant forces normal to the T cell surface induced calcium fluxes [81]. The Lang and Reinherz team performed further OT studies using step movement (instead of a sinusoidal movement) to apply force to the TCR–pMHC bonds, showing that calcium induction required up to four-orders of magnitude higher pMHC coating when the T cell was merely contacted the pMHC-bead without motion than when the T cell was moved relative to the bead to generate force on the TCR, with tangential motion to the T cell surface inducing higher calcium level than normal motion [85]. The authors also observe that the applied tangential force was relaxed by 8-nm steps that required actomyosin activity [85]. The ability for force to trigger calcium correlated with the rate of force relaxation, which, in turn, depended on the force direction. If force relaxed too slowly along one direction and did not trigger calcium, reversing the force to the opposite direction resulted in fast relaxation and calcium triggering.

Although it is not known the in vivo direction of force application on the TCR molecule, it may be reasonable to assume that both tensile and shear forces may apply. Even when the T cell is moved tangentially relative to pMHC-coated bead to which the cell contacts (as in some of the OT experiments), the force applied to the TCR–pMHC bond has more than merely a shear component perpendicular to the long axis of the bond, considering the highly irregular T cell surface microvilli at which TCRs localize and the likely pivotal motion of the bead about the bond (because the OT cannot prevent bead rotation on the cell surface) [4,6]. Several considerations may help explain the anisotropic data of the OT experiments [85,86]. A greater TCR tension would be generated by tangential than normal relative bead–cell displacement of the same magnitude [4]. Further, the TCR’s transmembrane and cytoplasmic anchor has to support both the normal and tangential force components resulted from moving the T cell tangentially relative to the OT-trapped bead but only the normal force component from a normal relative movement. Furthermore, the force on TCR is borne by its distinctive α and β chains through a diagonal docking interface with the pMHC. Neither molecule is axisymmetric about the TCR–pMHC long axis [90]. The one-sided coreceptors [91] (CD4 or CD8) and axially asymmetric CD3 subunits [90,92,93,94] further contribute to the asymmetry of the mechanical system. All may generate distinct effects when force is applied tangentially vs. normally.

Besides the magnitude and direction, other force parameters shown to have impacts on TCR triggering include the duration and frequency of force application, as well as step force vs. sinusoidal force and ramped force vs. clamped force types of force waveform. Clamping the force at constant values applies durable force to the TCR–pMHC bond, which seems to be important for TCR triggering, because applying linear ramp force until rupture does not induce calcium despite the higher force, magnitudes [81]. Interestingly, allowing CD8 to bind pMHC with the TCR cooperatively “rescues” such deficiency, enabling the ramp force to trigger calcium [95]. Noticeably, it was not only the prolongation of bond-lifetimes by catch-bonds that were requisite of calcium induction. Anti-TCR antibody formed a slip bond with much longer lifetime than the TCR bonds with agonist pMHC across all forces. Yet the calcium signals were strongest at 10 pN where the bond lifetime was shorter than those at lower forces [81]. By pooling all sequential binding events across time until the induction of calcium flux, it was found that the best predictor of calcium signal is an accumulated bond lifetime of 10 s within the first 60 s of TCR–pMHC engagements [81]. The idea that ligand discrimination occurs within minutes of engagement was corroborated when observing T cell spreading in an all-or-nothing and ligand affinity-dependent fashion [96].

A major group of TCR triggering models involves conformational changes. Whereas extensive crystallographic studies have not revealed sufficient amount of conformational changes in the ectodomains of the TCR–pMHC complex [90,91], convincing evidence indicates the occurrence of conformational changes in the CD3 cytoplasmic tails, which are released from the inner leaflet of the plasma membrane accompanying TCR triggering [97,98]. Force-induced conformational change has been suggested as a mechanism TCR triggering [99,100], e.g., by inducing apposition of the CD3ζζ subunits that are spread before TCR–pMHC binding [101] and/or segmenting the bipartite helix in the transmembrane domain of the TCR α-subunit to alter its association with the CD3 subunits [102], which may dislodge the CD3 tails from the plasma membrane. The Lang and Reinherz team was the first to observe a sudden length increase, or transition, in single TCR–pMHC [79] and pre-TCR–pMHC [39] bonds when OT was used to apply an external force on them. Interestingly, the transition distance increases with force, correlates with the peptide potency, and is reduced when the FG loop in the TCR Cβ region is deleted. Since the ΔFG mutant also suppressed TCR–pMHC [79] catch bond and the antibody H57 that binds an epitope near the FG loop induced a much more pronounced TCR–pMHC catch bond [79], the authors suggested that such conformational changes might occur at the TCR near the FG loop [79], and are important to TCR mechanosensing [102,103]. It is worth noting that measuring forces on bonds could benefit from an increase in throughput achievable by techniques that optically track resistance of dozens of particles or cells, in parallel, such as magnetic [104] or acoustic force spectroscopies [105]. However, currently there is a trade-off between the increased throughput and reduced sensitivity and resolution of these techniques, which has been discussed here to be important in observing events such as conformation changes.

Wu et al. performed molecular dynamics simulations and observed that the peptide-dependent conformational changes occurred in the MHC molecule as a result of force-induced dissociation of the intradomain bonds of the β_2m_ domain with the α_1_α_2_ and/or α_3_ domains [80]. Using mutagenesis to strengthen these interdomain interactions by disulfate bonds eliminated such conformational changes, as observed by direct single-molecule measurements using magnetic tweezers [80]. Importantly, these conformational changes underlie the TCR catch bonds with agonist pMHC, suggesting an allosteric mechanism similar to the sliding-rebinding mechanism observed for catch bonds of selectin–ligands [106], GPIbα–VWF [107] and actin–actin catch bonds [108]. Interestingly, these conformational changes are inhibited by cancer associated somatic mutations in the MHC, which suppresses catch bond formation, implicating a mechanism for cancer to escape immune surveillance [80]. Thus, allosteric changes in TCR–pMHC interactions may play a role in conferring the generation of catch bonds for ligand discrimination [80,97].

A recent study monitored the single-molecule Förster resonance energy transfer (FRET) between a Cy5-labeled single-chain variable fragment (scFv) of the antibody J1 bound to the TCR and a Cy3-labeled peptide bound to a MHC molecule (FRET1) and between an Alexa568-labeld scFv of another anti-TCR antibody J3 and a green fluorescent protein (GFP) attached to the C-terminus of the CD3ζ chain (FRET2) [78]. A 1D potential-of-mean-force (PMF) that trapped the TCR–pMHC in the bound state was calculated from the FRET1 signal distribution. The bottom location the PMF well (which was used as a proxy for bond length) and its steepness (which was related to the force required to pull the bond away from its equilibrium position by a unit distance, or bond strength) provide two metrics for the quality and the confirmation of the TCR–pMHC bond. The FRET2 signals were used to calculate the TCR-CD3ζ distance to gauge the degree of CD3ζζ tail swinging-out from the inner leaflet of the membrane bilayer. Interestingly, the length and strength of the TCR–pMHC bond as well as the TCR-CD3ζ distance all correlate with the peptide potency, with the CD3ζ phosphorylation, and with the intracellular calcium [78].

**Figure 3:**
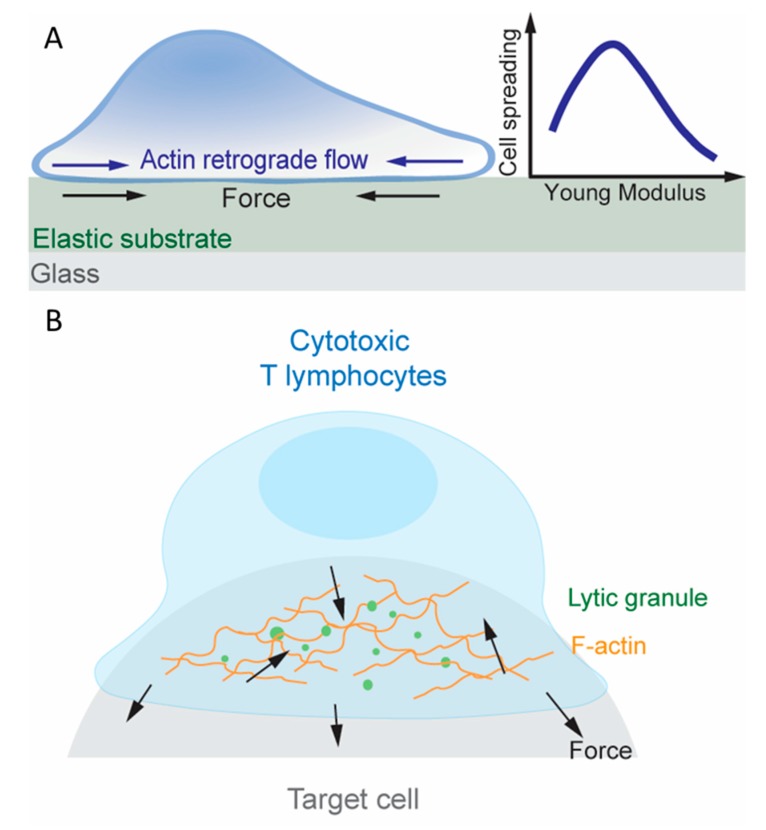
Deformation exerted by naïve T cells and cytotoxic lymphocytes. Schematics depicting how naïve and cytotoxic T cells deform their target substrates. (**A**) Cell spreading is most optimal when the Young’s modulus of the substrate is on the order of 10–100kPa. (**B**) Cytotoxic T lymphocytes (CTLs) spatially coordinate F-actin polymerization and lytic granule localization to mechanical hotspots where force can potentiate the release of granules.

Examination of dynamic bonds in the aforementioned studies relied on exertion of external forces. To visualize endogenous force, traction force microscopy (TFM) has been used besides the DNA-based TGT and MTP. Here, elastic substrates are embedded with beads that can be tracked under a microscope (Figure 3A) [109]. When cells deform the substrate, the traction force fields required to produce the observed bead displacements are determined by accounting for the known stiffness of the substrate. Another method for measuring cell-generated traction forces uses micropillar array detector (mPAD) whose deflection can be converted to traction force fields [110]. Note that, unlike TGT and MTP, which limits and reports, respectively, tension on the T cell surface molecules, TFM and mPAD reports the lateral component of traction forces on the substrate surfaces. In addition to the unreported normal components of force vectors, force waveforms experienced by individual receptors are difficult to translate from traction maps acquired through TFM or mPAD assays since their spatial distribution and potential for clustering within the two-dimensional junction is unknown. A related set of methods have been developed to study another aspect of mechanotransduction, namely, rigidity sensing, by measuring the T cells’ response to the changing stiffness of the elastic substrate and the micropillar assay [111]. These techniques have revealed extensive information about how T cells exert forces on their surface molecules (including TCR, coreceptor, co-stimulatory molecules and adhesion molecules), sense rigidity of the substrate through these molecules, and leverage mechanotransduction to promote activation [5].

**Figure 4:**
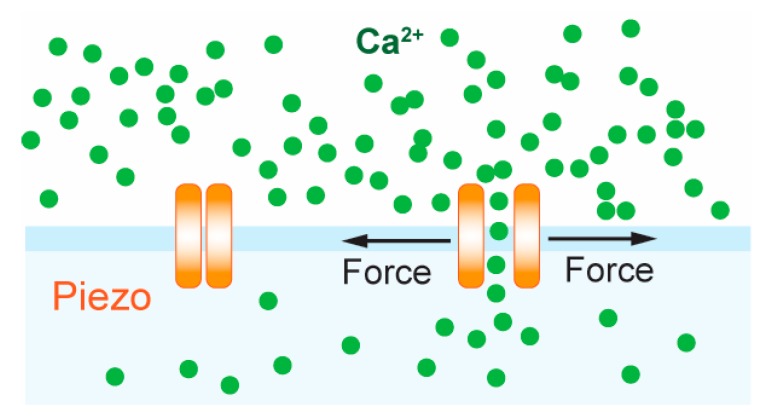
T cell activation by Piezo. Illustration depicting how force exerted on Piezo can induce entry of calcium flux resulting in T cell activation.

Although the TCR complex is proposed to be intrinsically mechanosensitive, recently, the professional mechanosensor Piezo1 was shown to enhance TCR triggering and activation [112]. Piezo1 is part of a conserved class of mechanically activated ion channels across eukaryote species, non-selectively permeable to cations, with a slight preference for Ca^2+^ ions [113,114]. It has to be shown to be involved in numerous processes such as vascular development [115], red blood cell volume regulation [116,117], bone formation [118], axonal growth [119] and lymphatic dysplasia [120]. Piezo1 has been found to be essential for innate immunity, allowing macrophages and monocytes to sense cyclical force in the lungs, inducing pro-inflammatory gene expression [121]. Chemical [112,122] and mechanical [123] stimulation of Piezo1 induced calcium flux in T cells and calcium-dependent nuclear factor of activated T-cells (NFAT) activation [123] (Figure 4). Piezo1-deficient CD4^+^ and CD8^+^ T cells showed impaired activation following bead-immobilized anti-CD3/anti-CD28 stimulation, as measured by ZAP-70 phosphorylation and CD69 expression, and impaired proliferation when stimulated with allogenic and autologous monocyte-derived dendritic cells. Moreover, Piezo1 knockdown abrogated Ca^2+^ flux in response to bead-immobilized anti-CD3 and anti-CD28 antibodies. Treatment with the agonist Yoda1, which can activate Piezo1 in the absence of mechanical stimuli, rescued T cell activation that was abolished by using soluble instead of surface bound anti-CD3/anti-CD28 antibodies, measured by ZAP-70 phosphorylation and CD69 expression [112]. In mice, T cells with GOF Piezo1 mutations (commonly present in African populations) were found to provide survival advantage during plasmodium infection, attenuating blood–brain barrier disruption and cerebral malaria [117].

In summary, dynamic bonds amplify pMHC discrimination leading to T cell activation and there is evidence that conformational changes in the TCR or the pMHC is responsible. Upon an accumulation of force-prolonged bonds, the T cell fluxes calcium indicative of activation. However, mechanical forces can also trigger activation through the mechanosensitive ion channel Piezo1. T cells that sense antigen exert forces, which may allow for further ligand discrimination toward specific and sensitive activation within minutes. These processes are dependent on the signaling of intracellular kinases and phosphatases. Overall, including mechanical considerations to TCR-pMHC binding has greatly enhanced our understanding of ligand discrimination and T cell activation.

## 7. Mechanotransduction in T Cell Effector Function

Following recognition of foreign antigen peptides loaded on APCs’ MHC molecules, T cells develop an organized IS consisting of TCR, costimulatory molecules [110] and adhesion molecules [124,125], which activate the T cell to proliferate and ultimately differentiate into helper T cells or cytotoxic T lymphocytes (CTLs) depending on their coreceptor, CD4 or CD8, respectively [11,126]. Altering the stiffness of the substrate on which pMHC, co-stimulatory molecules and/or adhesion molecules are presented impacts T cell proliferation and differentiation, indicating that T cells are capable of sensing the substrate rigidity via their surface receptors [111,127]. Rigidity sensing involves mechanotransduction and requires the cell to exert force to and to gauge the deformation of the substrate. Indeed, MTP studies showed that T cells pulled through the TCR and CD8 on pMHCs that were either immobilized on glass surfaces, which sustain lateral force [77], or reconstituted on supported lipid bilayers (SLPs), which do not sustain lateral force [128]. When plated on mPADs coated with anti-CD3, T cells applied lateral forces to deflect the micropillars [110]. When an antibody against the co-stimulatory molecule CD28 was also presented, the endogenous forces increased, suggesting a spatially modulated the cross-talk between the TCR and CD28 [110,129]. It has been reported that actin retrograde flow activates integrin α_L_β_2_ [130] within the IS and drive TCRs [131,132] to move towards the IS center where the central supramolecular activation cluster (cSMAC) resides. Traction forces were diminished when actin retrograde flow was suppressed by inhibiting actin polymerization, locking Arp2/3 to prevent actin branching, and destabilizing F-actin, highlighting the importance of the actin cytoskeleton in traction force generation [109]. The role of actin dynamics generated TCR force on T cell activation is also supported by the findings that preventing actin polymerization weakens calcium fluxes in T cells bound to pMHC coated on the AFM cantilever, and that applying exogenous forces on the TCR via the AFM cantilever to mimic the endogenous forces provided by actin retrograde flow rescues the calcium signals [87].

The IS is the junctional structure between a CD4^+^ helper T cell and B lymphocyte during targeted cytokine secretion, and a similar structure is also observed between a CTL and a target cell during the CTL’s killing of the virus infected or cancerous target cell, hinting at a reliance on mechanotransduction [133,134] (Figure 3B). CTL killing is exerted on an individual cell basis since it requires the polarization of the centrosome, which controls lytic granules, toward the site of TCR triggering, underscoring the specifically directed nature of exerting apoptosis and preventing damage to healthy tissue [135]. Indeed, the dynamics of CTL force exertion in the IS was recently measured directly using a newly developed superresolved microparticle TFM [136]. IS has been found to be a site of intense force exertion at the nanonewton scale and there existed a positive correlation between the stiffness of the target cell surface and the degree of cytotoxic granule release by CTLs [133,134,137]. Interestingly, release of lytic granules was spatio-temporally correlated with force exertion at the IS [138]. In a follow up study, Huse and colleagues investigated the spatio-temporal correlation between F-actin and the 3D structure of CTLs during lytic granule release [133]. F-actin enriched protrusions extended between micropillars that were coated with anti-CD3 or agonist pMHC and ICAM-1, well-separated from where the centrosome and lytic granule fusion occurred at the top of the micropillars, and these structures were sensitive to substrate rigidity [133,137]. The molecular and dynamic complexity of the IS confounds, which the molecule is acting as a mechanotransducer in the context of cytotoxic killing. However, aforementioned evidence suggests that force sensing through the TCR is critical for the cell-polarized formation of the centrosome where lytic granule release occurs.

While dynamic bonds and conformational changes were not observed in this system, these studies revealed that there is an optimal substrate for targeted cell killing that is dependent on stiffness and topology. Future work could study how the stiffness of virus-infected or transformed cells compare to established PDMS or hydrogel substrates with stiffnesses optimized to facilitate IS formation and centrosome localization.

## 8. Contraction and the Influence of the Microenvironment

Proliferation generates a large number of clonally expanded T cells to fight foreign intruders such as viruses. Once the viral threat is cleared, CTLs undergo contraction whereby most die by apoptosis. The timing by which different CTL clonotypes are released from lymph nodes and when they contract, however, is dependent on their respective TCR–pMHC interactions. Elegant experiments demonstrated that adoptively transferred OT1 cells underwent expansion in mice infected with *Listeria monocytogenes* expressing OVA faster than endogenous cells [12,139]. When the *L. monocytogenes* expressed variations of the OVA peptide with decreasing affinity for the OT1 TCR, the percentage of total CD8^+^ cells expressing that OT1 TCR at peak clonal expansion ranged from over 60% for WT OVA to about 1% for the weakest APL. Contraction of OT1 T cell numbers began after 7 days when the *L. monocytogenes* expressed strong APLs, after 6 days for intermediate APLs, and after 5 days for the weakest APL. These results underscore how a broad range of affinities, in this case up to 700-fold differences, is capable of activating T cells enough to undergo at least some level of clonal expansion. Furthermore, interaction with all APLs led to the development of functional memory T cells as seen with a plateau in the number of cells 15 days post infection, and expansion 4 days after secondary infection [139]. Thus, analogous to a panel of positive or negative selection ligands, there exists a panel of pMHCs for a single TCR that can lead to expansion and even differentiation into memory cells, albeit to different degrees. It should be cautioned however, that affinity-based measurements should be corroborated with force-imposed dynamic bond measurements since it has been recently demonstrated that force-free measurements may not predict the stimulatory degree of TCR–pMHC interactions [83]. Overall, TCR signaling lies at the center of the decisions of which T cells dominate the immune response.

Assuming derivation from single clones interacting with a specific antigen, clonal expansion of CTLs is still heterogeneous suggesting that extrinsic signals including intercellular communication and the microenvironmental niche may also regulate T cell functionality and fate [140]. To investigate the effects of these cues on T cell development, adoptively transferred T cells from P14 transgenic mice expressing a monoclonal TCR were examined in different anatomical niches of the spleen after the host mice were virally infected [13]. CD8^+^ P14 cells in the white pulp (WP) of the spleen and in the presence of host T-regulatory cells secreting TGF-β were observed to preferentially differentiate into memory cells, while those in the red pulp (RP) had a lower potential to differentiate the same way. Importantly, this observation was coincident with P14 T cells isolated from the WP having lower 2D affinity for the same pMHCtested against P14 T cells isolated from the RP during the early contraction phase. The higher affinity measurements of RP P14 cells were matched by functional outcomes as measured by specific target cell killing in vitro and viremia control in vivo. According to examination of genes related to membrane structure, T regulatory cells may provide extrinsic signals to cells in the WP to desensitize CTLs to antigen. The resulted lower affinity TCR–pMHC interactions may subsequently provide the appropriate amount of signaling to promote differentiation into memory T cells [13]. Thus, assuming that mechanotransduction is important in ligand discrimination as discussed in this review, the process may still be subject to shifts due to microenvironmental impositions. One such example suggested that nitration of tyrosines in TCR and CD8 impacted their interaction with pMHC [141]. Future studies will investigate how biochemical cues and intercellular interactions can interplay to influence TCR mechanotransduction. In particular, the ultra-structure of the T cell membrane could be an important determinant of TCR–pMHC binding [18,142]. Comprehensively understanding the role of TCR mechanotransduction in ensuring sensitivity and specificity of antigen recognition provides the platform to tackling questions that arise when the process of T cell activation shifts in disease states.

## 9. Conclusions

The decision of whether a T cell lives or dies is made at multiple junctures beginning from the moment a TCR β and α chain gene loci rearrange and are expressed on the surface membrane. TCR signals that surpass a certain threshold will result in the death of the clonotypes in order to reduce or prevent subsequent autoimmunity, whereas those that are not as strongly reactive are permitted to live and mature, expand into effector cells such as CTLs, initiate target cell killing, retract by apoptosis or become memory cells. All of these processes have been shown to depend on antigen recognition by the TCR, which relies on mechanical forces exerted by the T cell in order to amplify the signals necessary for the developmental progression. Despite the demonstrable role of mechanical forces in amplifying the TCR–pMHC signals, future work will further elucidate how the diversification of molecular mechanisms by which the same signaling cascade can induce survival, differentiation, function and death of the same T cell can shape the immune repertoire.
